# Supplementary material for: Quantitative Rapid Test for Detection and Monitoring of Active Pulmonary Tuberculosis in Nonhuman Primates
Source: Biology (Basel). 2021 Dec 2;10(12):1260. doi: 10.3390/biology10121260 (PMC8698365; doi:10.3390/biology10121260)
Supplement: Supplementary file 1 [file biology-10-01260-s001.zip › biology-1451355-supplementary.pdf]

SUPPLEMENTARY INFORMATION

QUANTITATIVE RAPID TEST FOR DETECTION AND MONITORING OF ACTIVE PULMONARY TUBERCULOSIS IN NONHUMAN PRIMATES

Zijie Zhou[a, 1], Anouk van Hooij[a, 1], Richard Vervenne[b], Claudia C. Sombroek[b], Elisa M. Tjon Kon Fat[c], Tom H. M. Ottenhoff[a], Paul L. A. M. Corstjens[c], Frank Verreck[b], and Annemieke Geluk[a,*]

Supplementary Table S1. ***Characteristics of NHP cohorts***

**Species**	**Cohort description**	**MTB dose**	**Number**	**Vaccination/ Treatment**	**Reference**
Macaca mulatta	High-dose MTB infection (Chinese genotype) rhesus macaques	500 CFU	23	BCG (n=11)<br>unvaccinated (n=12)	unpublished
Macaca mulatta	High-dose MTB infection Chinese type and Indian type rhesus macaques	500 CFU	30	BCG (n=18)<br>unvaccinated (n=12)	[1]
Macaca mulatta	Low-dose MTB infection RIF+INH treated (Indian genotype) rhesus macaques	15 CFU	18	RIF+INH (n=6)<br>- (n=12)	unpublished
Macaca mulatta<br>*Macaca fascicularis*	Ultra-low-dose MTB infection (Indian genotype) rhesus and cynomolgus macaques	1-7 CFU	20	- (n=20)	[2]

CFU: colony-forming unit; -, untreated; PA score, pathology score; RIF+INH, rifampin and isoniazid.

Supplementary Table S2. ***Characteristics of animals in the high-dose MTB infection rhesus macaque cohort***

Subject number	Sampling time point					Vaccination	Endpoint (weeks)	Humane endpoint	Total PA score	Lung PA score
1	wk37	wk3+	wk6+	wk12+	wk15+	-	15.1	Yes	84	40
2	wk37	wk3+	wk6+	wk12+	wk48+	-	51.0	No	29	6
3	wk37	wk3+	wk6+	wk12+		-	11.9	Yes	102	47
4	wk37	wk3+	wk6+	wk12+		-	13.0	Yes	96	46
5	wk37	wk3+	wk6+	wk12+		-	14.1	Yes	101	47
6	wk37	wk3+	wk6+	wk12+	wk15+	-	15.3	Yes	92	48
7	wk37	wk3+	wk6+	wk12+		-	12.1	Yes	100	46
8	wk37	wk3+	wk6+	wk12+	wk33+	-	34.0	Yes	74	37
9	wk37	wk3+	wk6+	wk12+	wk33+	BCG	33.4	Yes	111	50
10	wk37	wk3+	wk6+	wk12+	wk18+	BCG	16.9	Yes	100	49
11	wk37	wk3+	wk6+	wk12+	wk48+	BCG	50.4	No	22	9
12	wk37	wk3+	wk6+			-	14.1	Yes	104	45
13	wk37	wk3+	wk6+	wk12+		-	13.9	Yes	93	39
14	wk37	wk3+	wk6+			-	11.0	Yes	71	32
15	wk37	wk3+	wk6+	wk12+		-	12.0	Yes	46	18
16	wk37	wk3+	wk6+	wk12+	wk48+	BCG	49.7	No	11	4
17	wk37	wk3+	wk6+	wk12+	wk48+	BCG	49.7	No	31	5
18	wk37	wk3+	wk6+	wk12+	wk36+	BCG	35.7	Yes	63	22
19	wk37	wk3+	wk6+	wk12+	wk48+	BCG	51.1	No	22	2
20	wk37	wk3+	wk6+	wk12+	wk48+	BCG	50.4	No	34	7
21	wk37	wk3+	wk6+	wk12+	wk48+	BCG	49.9	No	49	14
22	wk37	wk3+	wk6+	wk12+	wk27+	BCG	28.9	Yes	26	5
23	wk37	wk3+	wk6+	wk9+		BCG	11.1	Yes	55	27

Rhesus macaques (n=23) were experimentally infected by endobronchial instillation with high-dose (500 CFU) MTB Erdman 38 weeks after vaccination with BCG. Sampling time points are relative to BCG vaccination, while '+' indicates time points relative to MTB infection. CFU, colony-forming unit; -, unvaccinated; PA score, pathology score; wk, week.

Table S3. ***Characteristics of animals in the high-dose MTB infection cohorts of Chinese type and Indian type rhesus macaques.***

Subject	Type	Vaccination	Endpoint (weeks)	Humane endpoint	Total PA score	Lung PA score
**1**	CHN	-	13.0	No	25	5
**2**	CHN	-	13.0	No	96	34
**3**	CHN	-	12.6	No	47	33
**4**	CHN	-	12.6	No	31	12
**5**	CHN	-	11.6	No	33	15
**6**	CHN	-	11.6	No	77	29
**7**	CHN	BCG i.d.	12.4	No	31	4
**8**	CHN	BCG i.d.	12.4	No	63	29
**9**	CHN	BCG i.d.	12.0	No	65	32
**10**	CHN	BCG i.d.	12.0	No	47	23
**11**	CHN	BCG i.d.	11.3	No	70	36
**12**	CHN	BCG i.d.	11.3	No	9	4.5
**13**	CHN	BCG muc.	13.0	No	67	29
**14**	CHN	BCG muc.	13.0	No	12	4
**15**	CHN	BCG muc.	12.1	No	21	5
**16**	CHN	BCG muc.	12.1	No	30	8
**17**	CHN	BCG muc.	11.4	No	28.5	4.5
**18**	CHN	BCG muc.	11.4	No	37	11
**19**	IND	-	8.0	Yes	97.5	43
**20**	IND	-	12.4	No	73	36
**21**	IND	-	11.0	Yes	89	49
**22**	IND	-	12.0	No	73	54
**23**	IND	-	11.6	No	118	62
**24**	IND	-	11.6	No	59	25
**25**	IND	BCG i.d.	12.4	No	66	33
**26**	IND	BCG i.d.	12.4	No	52	29
**27**	IND	BCG i.d.	12.0	No	14	5
**28**	IND	BCG i.d.	12.0	No	41	8
**29**	IND	BCG i.d.	11.3	No	69	43
**30**	IND	BCG i.d.	11.3	No	56	32

BCG-vaccinated Chinese-type rhesus macaques (CHN; n=18) and Indian-type rhesus macaques (IND; n=12) and unvaccinated control macaques were infected with high-dose (500 CFU) MTB Erdman.

-, unvaccinated; i.d., intradermal; muc., pulmonary mucosal (by endobronchial instillation); CFU, colony-forming unit; PA score, pathology score.

Table S4. ***Characteristics of animals in the low-dose MTB infection RIF+INH treated rhesus macaque cohort***

Subject	Treatment	Endpoint (weeks)	Humane endpoint	Total PA score	Lung PA score
**1**	RIF+INH	13	No	4	4
**2**	RIF+INH	13	No	13.5	5.5
**3**	RIF+INH	13	No	16.5	4.5
**4**	RIF+INH	13	No	7	5
**5**	RIF+INH	13	No	5	5
**6**	RIF+INH	9	Yes	16	7
**7**	-	11	No	35.5	18.5
**8**	-	9	No	16	3
**9**	-	8	Yes	107.5	57
**10**	-	11	No	20.5	7
**11**	-	7	No	57.5	18.5
**12**	-	7	No	32	9
**13**	-	11	No	27.5	4
**14**	-	11	No	34.5	18.5
**15**	-	9	No	46.5	26
**16**	-	11	No	72.5	36
**17**	-	11	No	46	18.5
**18**	-	10	Yes	74	49.5

Rhesus macaques (n=18) were infected with low-dose (15 CFU) MTB Erdman, six of the rhesus macaques received RIF+INH treatment from week 4 to week 12.

INH, isoniazid; RIF, rifampin; -, no antibiotics; CFU, colony-forming unit; PA score, pathology score.

Table S5. Characteristics of animals in ultra-low-dose *MTB* infected rhesus and cynomolgus macaque cohort

Subject number	Species	Endpoint (weeks)	Humane endpoint	Total PA score	Lung PA score
1	Cynomolgus	12	No	21.0	4.5
2	Cynomolgus	6	No	12.0	4.0
3	Cynomolgus	12	No	11.5	9.0
4	Cynomolgus	6	No	10.5	3.0
5	Cynomolgus	12	No	40.0	18.0
6	Cynomolgus	6	No	16.0	3.0
7	Cynomolgus	6	No	25.0	21.5
8	Cynomolgus	6	No	11.5	0.0
9	Cynomolgus	12	No	1.0	1.0
10	Cynomolgus	6	No	16.0	6.5
11	Rhesus	6	No	28.5	22.0
12	Rhesus	6	Yes	56.5	50.5
13	Rhesus	12	No	35.0	22.0
14	Rhesus	12	No	36.0	28.0
15	Rhesus	6	No	13.5	8.0
16	Rhesus	6	No	14.0	10.0
17	Rhesus	6	No	11.5	7.5
18	Rhesus	6	No	17.5	12.5
19	Rhesus	12	No	28.5	15.5
20	Rhesus	12	No	12.5	7.0

Rhesus macaques (n=10) and cynomolgus (n=10) macaques were infected with low-dose (1-7 CFU) MTB Erdman.

CFU, colony-forming unit; PA score, pathological score.

Table S6. Homology among proteins or nucleotide of biomarkers in rhesus macaques and marmosets with humans.

a. Nucleotide sequences alignment of biomarkers in rhesus macaques and marmosets with human

Biomarker	Official symbol (official full name) in NCBI	Rhesus macaques (*Macaca mulatta*)				White-tufted-ear marmoset (*Callithrix jacchus*)		
		UCP-LFA performance		Sequence alignment		UCP-LFA performance	Sequence alignmen	
				Query cover (%)	Percent identity (%)		Query cover (%)	Percent identity (%)
CCL4	CCL4 (C-C motif chemokine ligand 4) *	no signal		100	91.01	-		
IL-1Ra	IL1RN (interleukin 1 receptor antagonist)			48	92.79	-		
TNF	TNF (tumor necrosis factor)			93	92.94	-		
Ferritin	FTL (ferritin light chain)			92	94.44	-		
	FTH1 (ferritin heavy chain 1)			90	91.62	-		
S100A12	S100A12 (S100 calcium binding protein A12)			46	93.78	-		
CRP	C-reactive protein			93	92.32	no signal	98	84.82
ApoA1	APOA1 (apolipoprotein A1)	low signal	no different between HE & EE	100	94.27	-		
C1q	C1QA (complement C1q A chain)			96	92.37	-		
	C1QB (complement C1q B chain)			97	92.38	-		
	C1QC (complement C1q C chain)			99	92.52	-		
IL-6	IL6 (interleukin 6)		include	76	95	no signal	73	89.86
SAA1	SAA1 (serum amyloid A1)	high signal		96	91.38		91	84.43
IP-10	CXCL10 (C-X-C motif chemokine ligand 10)			63	92.95		99	87.92

b. Protein sequences alignment of biomarkers in rhesus macaques and marmosets with human

Biomarker	Protein name in NCBI	Human	Rhesus macaques (*Macaca mulatta*)					White-tufted-ear marmoset (*Callithrix jacchus*)			
		GI	UCP-LFA performance		GI	Sequence alignment		UCP-LFA performance	GI	Sequence alignmen	
						Query cover (%)	Percent identity (%)			Query cover (%)	Percent identity (%)
CCL4	C-C motif chemokine 4	49457234	no signal		1674557684	100	91.3	-			
IL-1Ra	interleukin-1 receptor antagonist protein	1827280156			109104278	100	95.48	-			
TNF	tumor necrosis factor	1159611449			114051684	100	96.14	-			
ferritin	ferritin heavy chain	56682959			355567690	100	94.65	-			
	ferritin light chain	20149498			386781446	100	97.14	-			
S100A12	protein S100-A12	5032059			966920425	100	86.96	-			
CRP	C-reactive protein	17975775			1622829517	99	91.48	no signal	675744606	99	85.27
ApoA1	Apolipoprotein A-I	13529242	low signal	no different between HE & EE	1622866776	100	95.13	-			
C1q	complement C1q subcomponent subunit A	1109457904			966915375	100	91.02	-			
	complement C1q subcomponent subunit B	87298828			966915377	100	95.26	-			
	complement C1q subcomponent subunit C	1109663887			388453100	100	96.33	-			
IL-6	interleukin-6	10834984		include	418203918	100	96.7	no signal	110180456	100	89.15
SAA1	SAA1	13937839	high signal		1622863919	100	87.7	no signal	532441807	100	85.25
IP-10	CXCL10	15012099			74136347	100	95.92	no signal	390460731	98	79.38

Homology among proteins or nucleotide of host serum proteins SAA1, IP-10, IL-6, CCL4, IL-1Ra, TNF, ferritin, S100A12, CRP, ApoA1, and C1q in rhesus macaques (*Mcaca mulatta*) and marmosets (*Callithrix jacchus*) with humans (*homo sapiens*) were calculated by Nucleotide BLAST and Protein BLAST. Protein levels were measured by UCP-LFA in serum from different dose MTB Erdman infected rhesus macaques (500 CFU, n=11; Figure S1) and marmoset (10, 50, 250 CFU, n=26; data not shown). Nucleotide **(a)** and protein **(b)** sequences were queried from Gene Database and Protein Database in NCBI and calculated sequence alignment by Nucleotide BLAST and Protein BLAST. NCBI, National Center for Biotechnology Information; *, CCL4 (C-C motif chemokine ligand 4) and CCL4L1 (C-C motif chemokine ligand 4-like 1) gene were compared in human and rhesus macaques, respectively; -, not performed; GI, GenInfo Identifier.

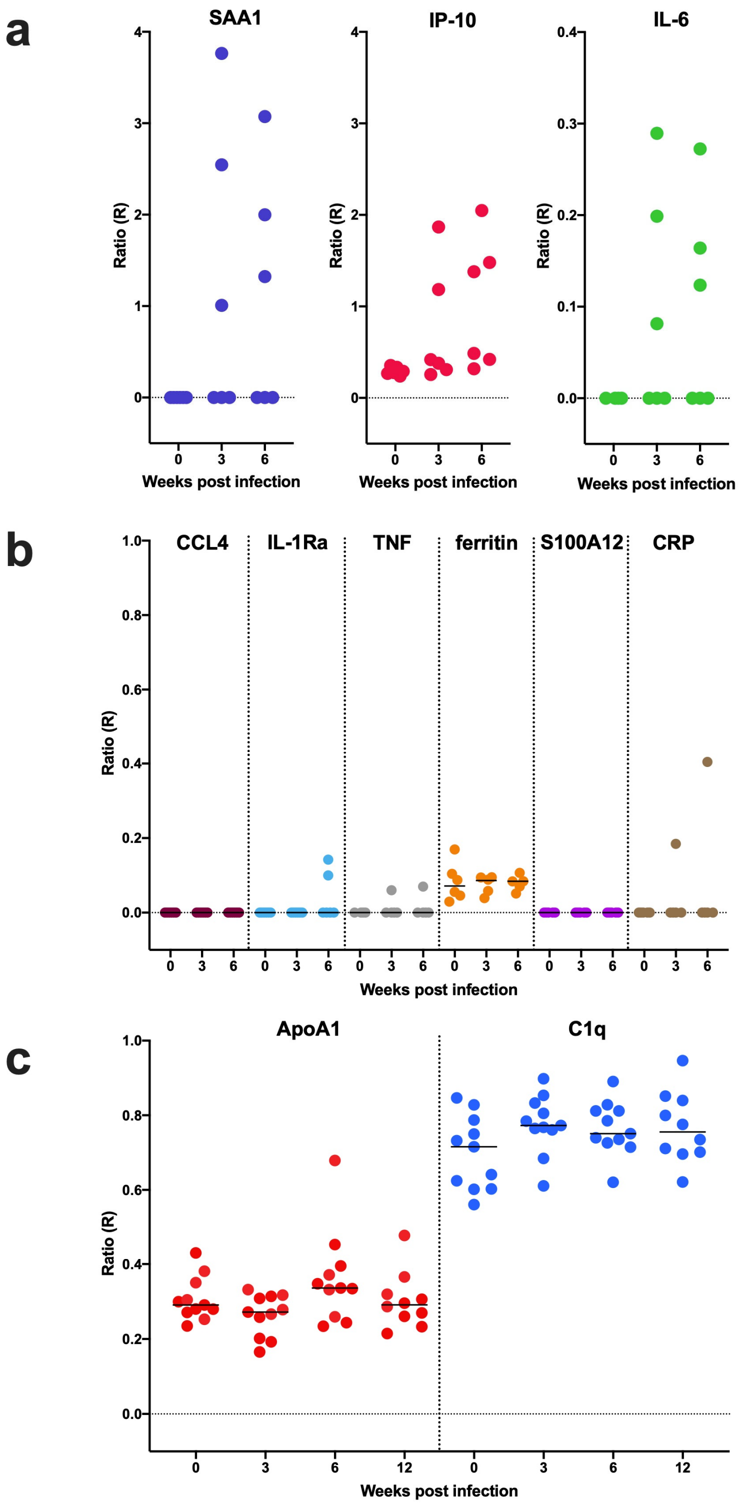

Figure S1: Assessment by UCP-LFA of 11 serum proteins as putative markers of experimental TB infection in rhesus macaques. Host serum proteins SAA1, IP-10, IL-6, CCL4, IL-1Ra, TNF, ferritin, S100A12, CRP, ApoA1, and C1q levels were

measured by UCP-LFA in serum from high-dose (500 CFU) MTB Erdman infected rhesus macaques (**a**, **b**, n=6; **c**, n=11). Results are displayed as the Ratio value (R) between Test (T) and Flow-Control (FC) signal based on relative fluorescence units (RFUs; excitation at 980nm and emission at 550 nm) measured at the respective lines (*y*-axis). The median values observed at each timepoint (*x*-axis) are indicated by horizontal lines. Each marker is indicated with a different color. Significant differences between HE versus EE at each timepoint were determined by Mann-Whitney U. **(a)** Levels of SAA1, IL-6, and IP-10 were increased after infection. **(b)** CCL4, IL-1Ra, TNF, S100A12, and CRP, were not detected in any of the serum samples, while ferritin was detected in very low concentrations (median <0.086). **(c)** C1q and ApoA1 levels did not show significant differences between the four timepoints.

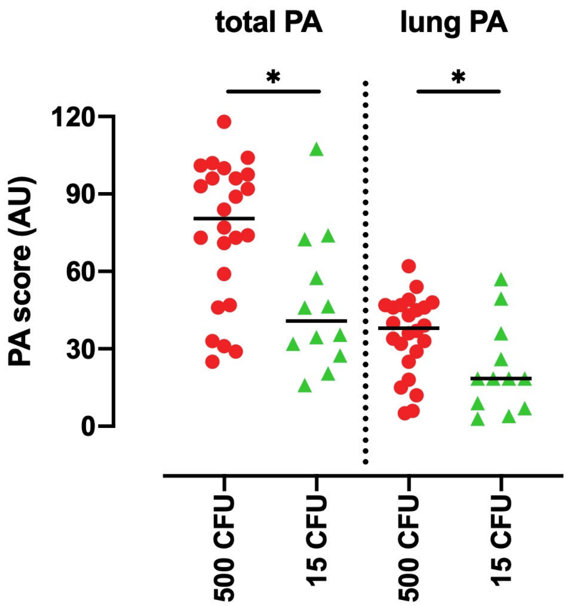

Figure S2. TB disease manifestation in rhesus macaques. Rhesus macaques (n=36) were infected with a single high-dose of 500 CFU (n=24, red dots) or low-dose of 15 CFU (n=12, green triangles) MTB Erdman. Tuberculosis pathology (PA; *y*-axis) was quantified post-mortem using an arbitrary, semi-quantitative grading system for the size, manifestation, and number of lesions, and for lymph node involvement, expressed in arbitrary units (AU). The median values of each group are indicated by horizontal lines. TB disease manifestation was more severe in the high-dose infected macaques. Significant differences between high- versus low-dose were determined by Mann-Whitney U tests. P-values: *$p<0.05$.

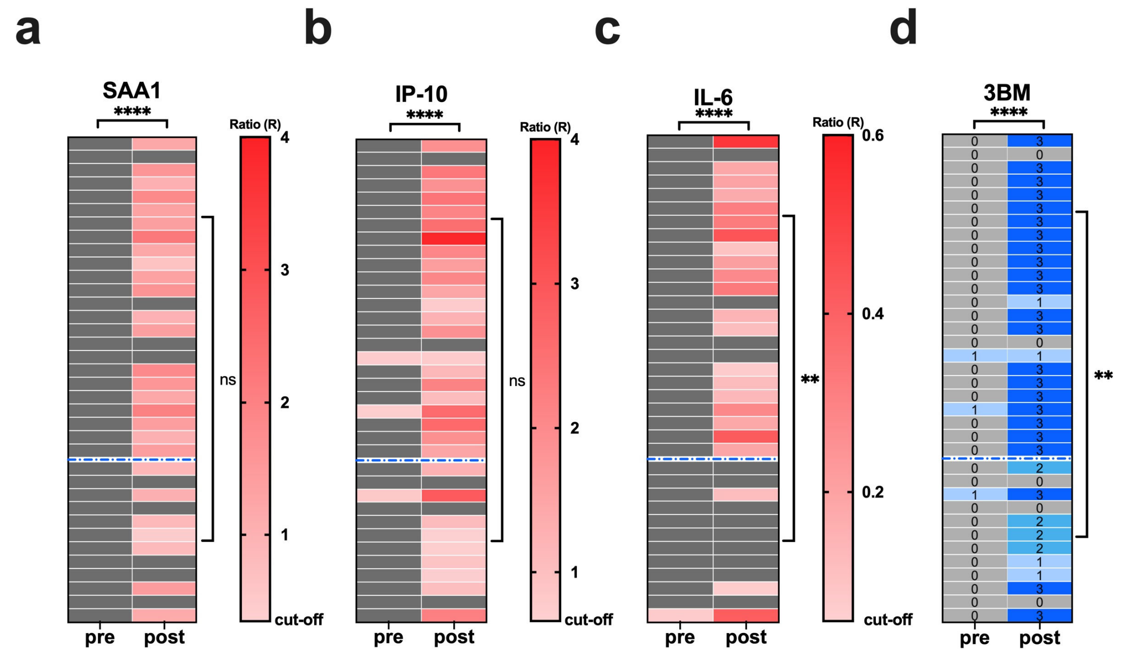

e

	Proportion of positive in high-dose group post-infection (%)	Proportion of positive in low-dose group post-infection (%)	P value
SAA1	83.33	58.33	0.1256
IP-10	91.67	75.00	0.3074
IL-6	83.33	25.00	<0.01

Figure S3: Heatmap showing SAA1, IP-10 and IL-6 levels of each rhesus macaque pre- and post-*MTB* infection. SAA1, IP-10, and IL-6 levels were measured by UCP-LFA in serum from high-dose (500 CFU, n=24, above the blue dotted line) or low-dose (15 CFU; n=12, below the blue dotted line) MTB Erdman infected rhesus macaques (RM) pre-infection, and at the endpoint post-infection. Each square represents one animal either pre-infection (left) or post-infection (right). Per biomarker above the cut-off value for each biomarker were considered positive. Cut-

off applied was described in Table 1b. The ratios lower than cut-off values are indicated in grey. (**a, b, c**) Heatmap of the SAA1, IP-10, IL-6 levels. The statistical significance in the proportion of positives was calculated by Fisher's exact test and shown in **table e**. (**d**) Heatmap for the outcome of the 3BM (3 biomarkers) signature was generated using the sum of all positive tests results from the individual markers. The significant differences were determined by Mann-Whitney U tests. The statistical significance level observed between pre- and post-infection is indicated at the top of the heatmaps, the differences between high- and low-dose are indicated on the right side of the heatmap. RM. P-values: **$p<0.01$, ****$p<0.0001$; ns, not significant.

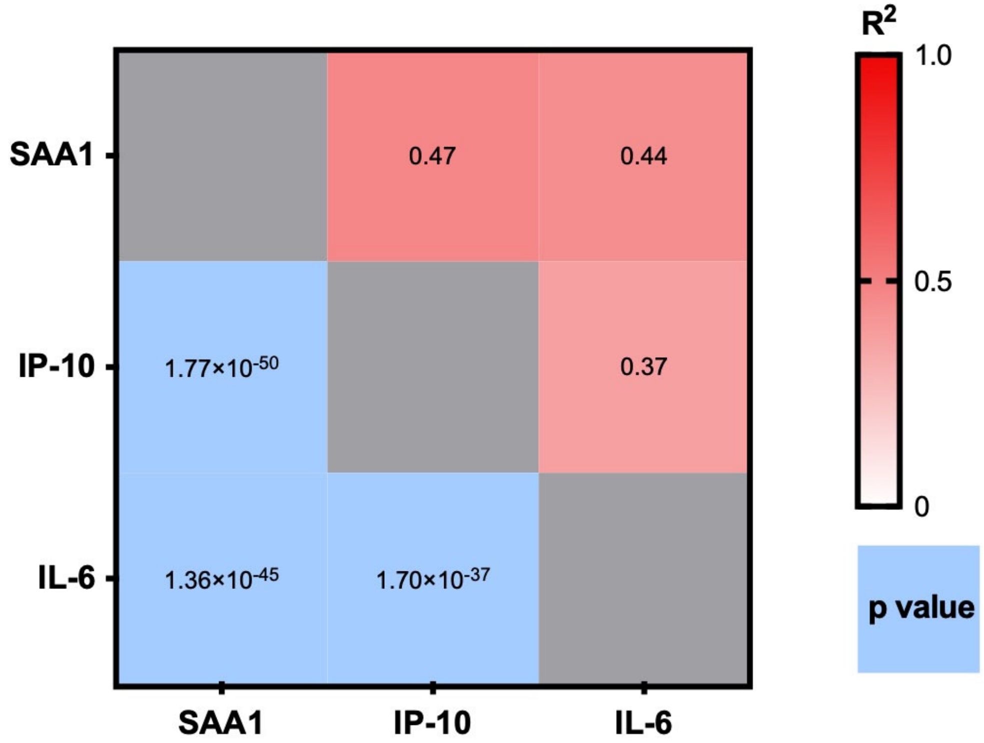

Figure S4: Correlation between three biomarkers. SAA1, IP-10, and IL-6 levels were measured by UCP-LFA in all serum samples (n=352) from MTB Erdman (500 CFU, n=53; 15 CFU, n=12; <7 CFU, n=10) infected but untreated rhesus macaques (n=75) in biobank. R^2 values, the square of the Spearman correlation coefficients, are depicted in red. The corresponding p-values, indicating the significance level of the observed correlation between the levels of the three biomarkers, are shown in blue.

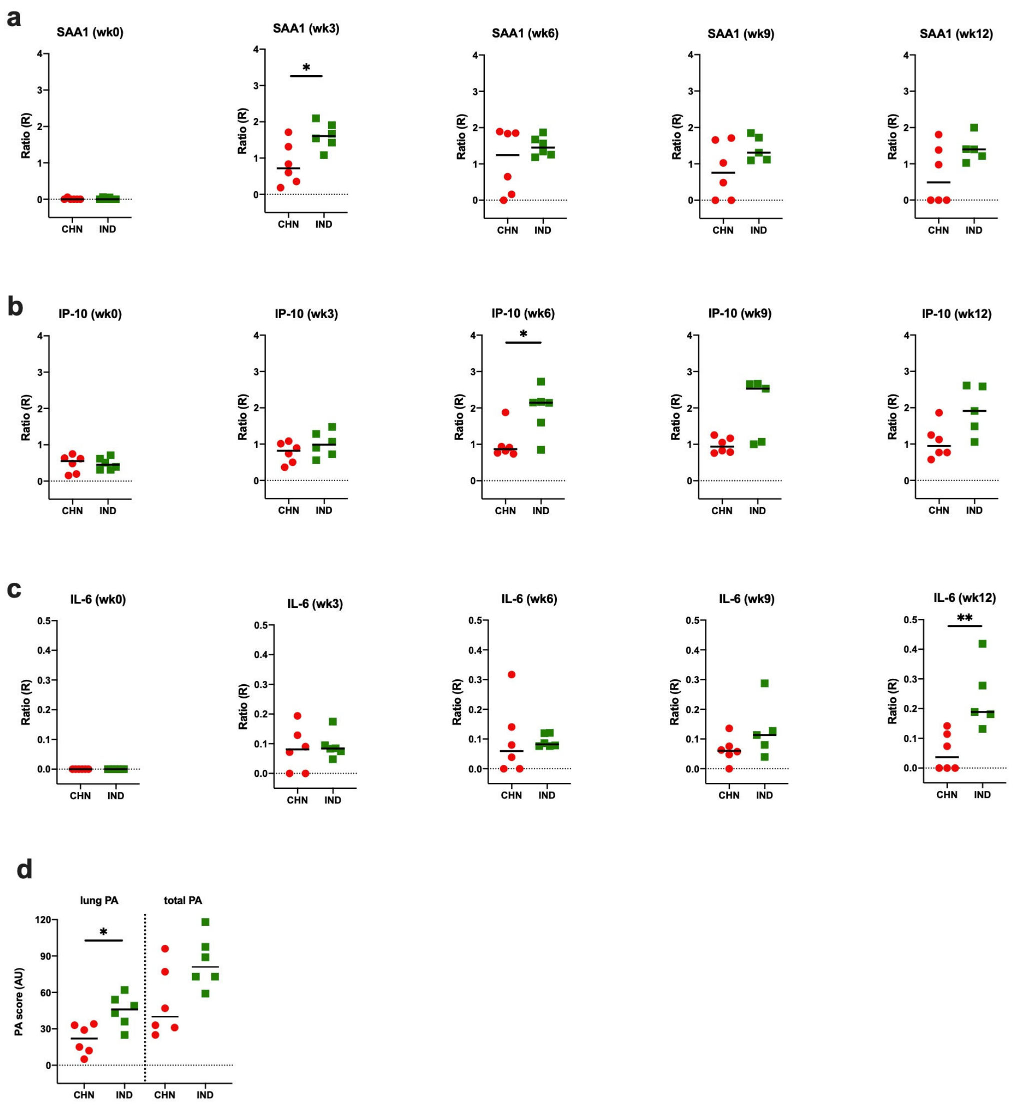

Figure S5. **SAA1, IP-10 and IL-6 levels in two rhesus genotypes that differ in disease susceptibility.** SAA1, IP-10 and IL-6 levels were measured by UCP-LFA in serum from high-dose (500 CFU) MTB Erdman infected Chinese-type (CHN, n=6, red dot) and Indian-type (IND, n=6, green square) rhesus macaques. (Since one Indian-type rhesus macaques reached a humane endpoint before week 12, only 5 measurements are depicted at wk12 in the IND group). The median values of each group are indicated by horizontal lines. (**a, b, c**) SAA1, IP-10 and IL-6 levels (*y*-axis) were significantly increased post-infection in both types of rhesus macaques. (**d**) Tuberculosis pathology

(PA; *y*-axis) was quantified post mortem using an arbitrary, semi-quantitative grading system for the size, manifestation, and number of lesions, and for lymph node involvement, expressed in arbitrary units (AU). Chinese-type have significantly lower lung PA scores than Indian type rhesus macaques as previously described[1]. Significant differences between CHN versus IND were determined by Mann-Whitney U tests. P-values: *p<0.05, **p<0.01.

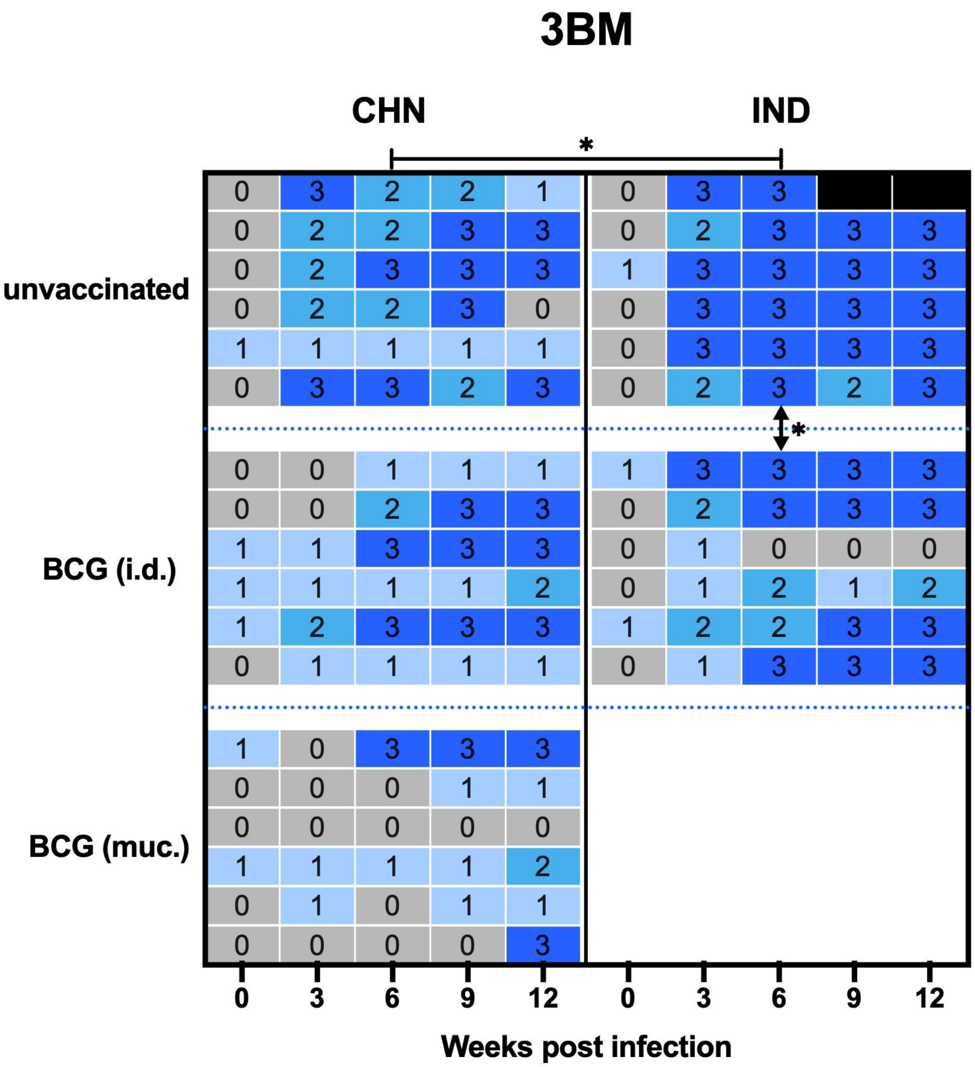

Figure S6: Heatmap for 3 biomarker signatures for TB in two genotypically different rhesus macaques. SAA1, IP-10 and IL-6 levels were measured by UCP-LFA in serum of high-dose (500 CFU) MTB Erdman infected Chinese type (CHN, n=18, left panel) and Indian type (IND, n=12, right panel) rhesus macaques. 12 macaques were unvaccinated (CHN, n=6; IND, n=6; upper panels), and 18 vaccinated with BCG by intradermal injection (BCG (i.d.) for both CHN (n=6) and IND (n=6) rhesus macaques; middle panels) or pulmonary mucosal administration (BCG (muc.), for the CHN rhesus macaques only (n=6), lower panel) 17 weeks before infectious

challenge with MTB. Per biomarker values above cut-off were considered positive. Cut-off values are described in Table 1b. Heatmap for the outcome of the 3BM (3 biomarkers) signature was generated using the sum of all positive tests results from the individual markers. In case of lack of data due to a premature humane endpoint, cells are indicated in black. The significant differences were determined by Mann-Whitney U tests and indicated by capped line (CHN vs IND) or two-head arrow (vaccinated versus unvaccinated) with an asterisk (*) for $p < 0.05$.

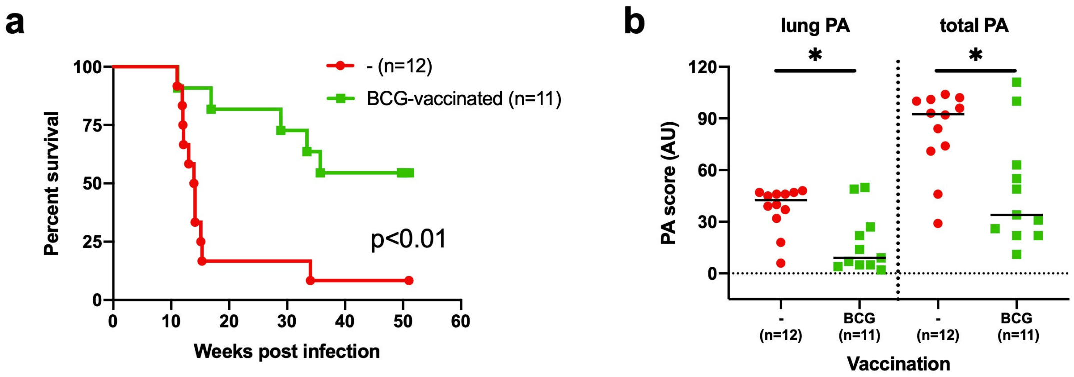

Figure S7. TB disease manifestation in rhesus macaques. Unvaccinated (n=12, green square) and BCG-vaccinated (n=11, red dot) rhesus macaques were infected with a single high-dose of 500 CFU of MTB Erdman. (**a**) Kaplan-Meier curve of survival time is significantly extended after prior BCG vaccination. (**b**) Tuberculosis pathology (PA; *y*-axis) was quantified post-mortem using an arbitrary, semi-quantitative grading system for the size, manifestation, and number of lesions, and for lymph node involvement, expressed in arbitrary units (AU). Lung and total pathology scores were significantly reduced by BCG vaccination prior to MTB infection. The median values of each group are indicated by horizontal lines. Significant differences between survival curves were determined by Mantel-Cox test; differences between unvaccinated versus BCG-vaccinated were determined by Mann-Whitney U tests. P-values: *p<0.05. – indicates unvaccinated.

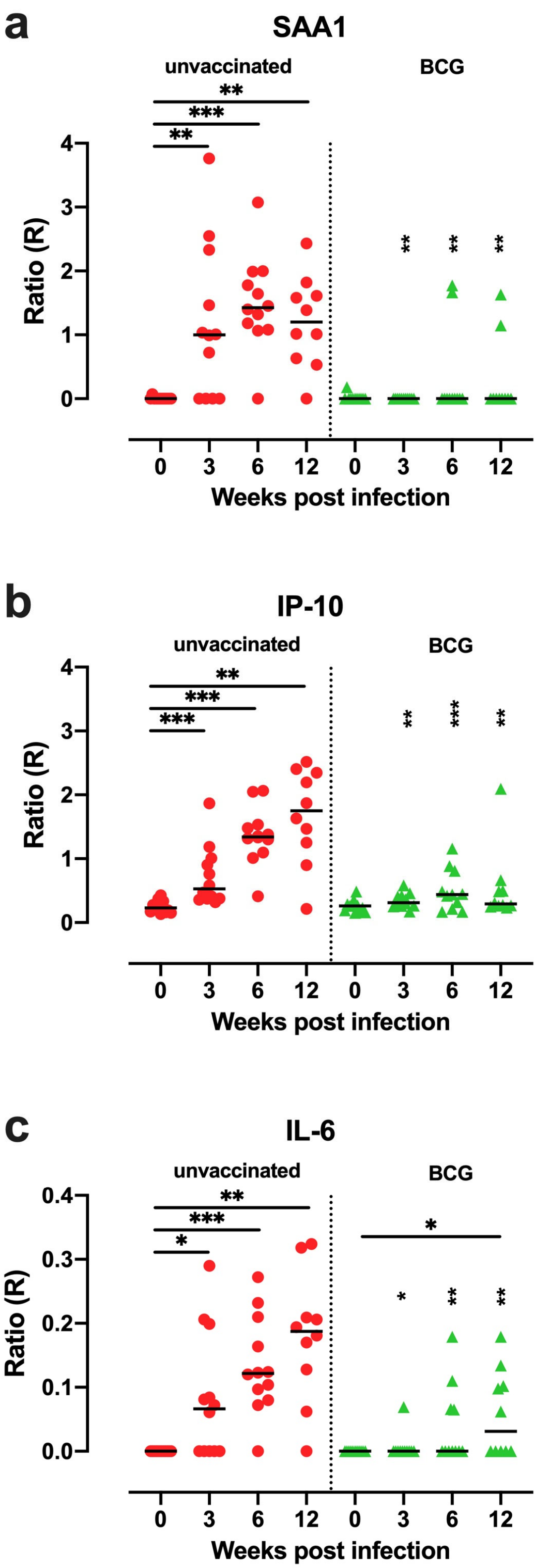
a
SAA1
unvaccinated
BCG
Ratio (R)
Weeks post infection
b
IP-10
unvaccinated
BCG
Ratio (R)
Weeks post infection
c
IL-6
unvaccinated
BCG
Ratio (R)
Weeks post infection

Figure S8. BCG vaccination decreases SAA1, IP-10 and IL-6 levels in serum after *MTB* infection. SAA1, IP-10 and IL-6 levels (*y*-axis) were measured by UCP-LFA in serum from (high-dose) MTB Erdman infected rhesus macaques (n=23). Red dots indicate unvaccinated macaques (n=12), green triangles indicate BCG-vaccinated macaques (n=11). (Since two unvaccinated macaques reached a humane endpoint before week 12, only 10 measurements are depicted at week 12 in the unvaccinated group). The median values of each group are indicated by horizontal lines. **(a, b, c).** The levels of SAA1, IP-10 and IL-6 in unvaccinated macaques at week 3, 6 and week 12 post-infection were higher than the BCG vaccinated macaques. Significant differences between each timepoint versus week 0 were determined by Wilcoxon matched-pairs tests (Since two unvaccinated macaques reached a humane endpoint before week 12, only 10 pairs were compared on week 12); differences between unvaccinated versus BCG-vaccinated were determined by Mann-Whitney U tests indicated in the right panel (BCG). P-values: *p<0.05, **p<0.01, ***p<0.001.

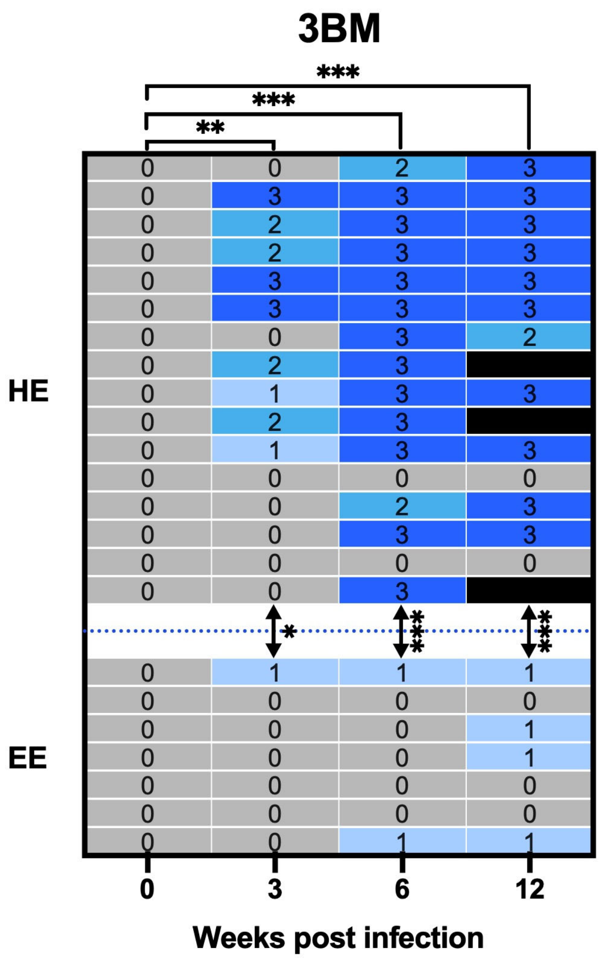

Figure S9: Heatmap for 3 biomarker signatures for TB in rhesus macaques with different disease severity. SAA1, IP-10 and IL-6 levels were measured by UCP-LFA in serum from high-dose (500 CFU) MTB Erdman infected rhesus macaques (n=23), of which 16 animals developed progressive disease and reached a premature humane endpoint (HE) while the endpoint by protocol was fixed at 50 weeks post-challenge (EE). (Since three rhesus macaques reached a humane endpoint before week 12, only 13 at wk12 in the HE group). Per biomarker values above cut-off were considered positive. Cut-off values are described in Table 1b. Heatmap for the outcome of the 3BM (3 biomarkers) signature was generated using the sum of all positive tests results from the individual markers. In case of lack of data due to a premature humane

endpoint, cells are indicated in black. The significant differences were determined by Mann-Whitney U tests and indicated by capped line with p-value (between time points) or two-head arrow with p-value (HE versus EE). P-values: *$p<0.05$, **$p<0.01$, ***$p<0.001$.

REFERENCES:

1. Verreck, F.A.W., et al., *Variable BCG efficacy in rhesus populations: Pulmonary BCG provides protection where standard intra-dermal vaccination fails.* Tuberculosis (Edinb), 2017. **104**: p. 46-57.
2. Dijkman, K., et al., *Disparate Tuberculosis Disease Development in Macaque Species Is Associated With Innate Immunity.* Frontiers in Immunology, 2019. **10**: p. 18.
